# Ameloblastic Fibroodontoma of the Mandible with Normal Karyotype in a Pediatric Patient

**DOI:** 10.1155/2012/969687

**Published:** 2012-08-09

**Authors:** Esther Manor, Elena Kan, Lipa Bodner

**Affiliations:** ^1^Institute of Human Genetics, Soroka Medical Center, and Faculty of Health Sciences, Ben-Gurion University of the Negev, Beer-Sheva 84105, Israel; ^2^Department of Pathology, Soroka Medical Center, and Faculty of Health Sciences, Ben-Gurion University of the Negev, Beer-Sheva 84105, Israel; ^3^Department of Oral and Maxillofacial Surgery, Soroka Medical Center, and Faculty of Health Sciences, Ben-Gurion University of the Negev, P.O. Box 151, Beer-Sheva 84105, Israel

## Abstract

*Background*. Ameloblastic fibroodontoma (AFO) is a rare mixed odontogenic tumor with epithelial and mesenchymal components. AFO presents as a painless swelling in the mandible or maxilla. Radiographs show a well-defined radiolucent area containing various amounts of radiopaque material of irregular size and form. The common treatment is enucleation. It is not an aggressive tumor but recurrence and malignant transformation are possible. *Methods*. An AFO of the mandible of a 3-year-old female is reported. Panoramic radiograph and CT scan revealed a unilocular lesion with radiopaque center and radiolucent margins. Enucleation was performed with a good outcome. *Results*. Histopathology was a classic AFO. The karyotype was normal. No recurrence was noted at 12 months. *Conclusions*. As it is a benign tumor with low recurrence rate, conservative surgery is the treatment of choice. As malignant transformation to ameloblastic fibrosarcoma or ameloblastic odontosarcoma is possible despite the normal karyotype, long-term followup is recommended.

## 1. Introduction

Head and neck tumors rarely affect children, but odontogenic tumors (OT) are are frequent among children and adolescents. The main types of OT are ameloblastoma (AME) and odontoma (ODO). Ameloblastic fibro-odontoma (AFO) is a rare mixed OT with epithelial and mesenchymal components. It is usually diagnosed between the first and the second decades, with no clear gender predilection.

Clinically, it presents as a painless swelling of the posterior mandible or maxilla, associated with unerupted teeth. Radiographically, a well-defined radiolucent area containing various amounts of radiopaque material of irregular size and form with density resembling dental tissue is seen [1, 2].

The World Health Organization (WHO) classification defines AFO as a neoplasm composed of odontogenic epithelium embedded in cellular ectomesenchymal tissue that resembles dental papilla, with varying degrees of inductive change and dental hard tissue formation [3]. We are unaware on a cytogenetic workup of AFO in a pediatric patient.

The purpose of this paper is to report on a case of AFO occurring in the mandible of a 3-year-old female patient, its diagnosis, cytogenetic, surgical management, and outcome.

## 2. Case Report

A 3-year-old female presented with a swelling on her face noticed by her parents. Exraoral examination revealed a mild facial asymmetry and a swelling of the left mandible. Oral examination showed a firm, smooth swelling of the left mandibular mucosa. All the deciduous teeth were intact.

**Figure 1:**
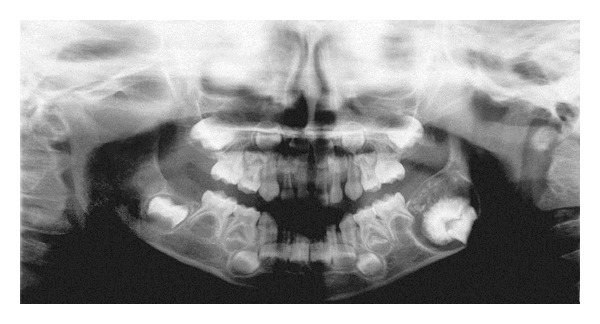
Preoperative panoramic radiograph revealed a unilocular radiolucent lesion with scattered radiopaque foci in the center. The first permanent mandibular molar is displaced toward the inferior border of the mandible.

**Figure 2:**
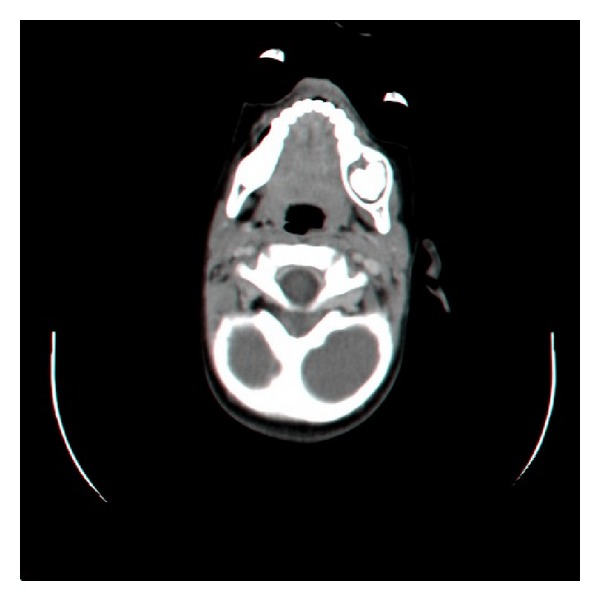
Axial CT of the mandible demonstrates a radiolucent-radiopaque lesion with well-defined borders affecting the left body of the mandible.

A panoramic radiograph revealed a unilocular radiolucent lesion with scattered radiopaque foci mainly in the center. The first permanent mandibular molar is displaced inferiorly (Figure 1). Axial CT scan of the mandible revealed a unilocular well-circumscribed radiolucent lesion with radiopaque center of the posterior left mandible (Figure 2). The clinical impression was of an odontogenic lesion. 

**Figure 3:**
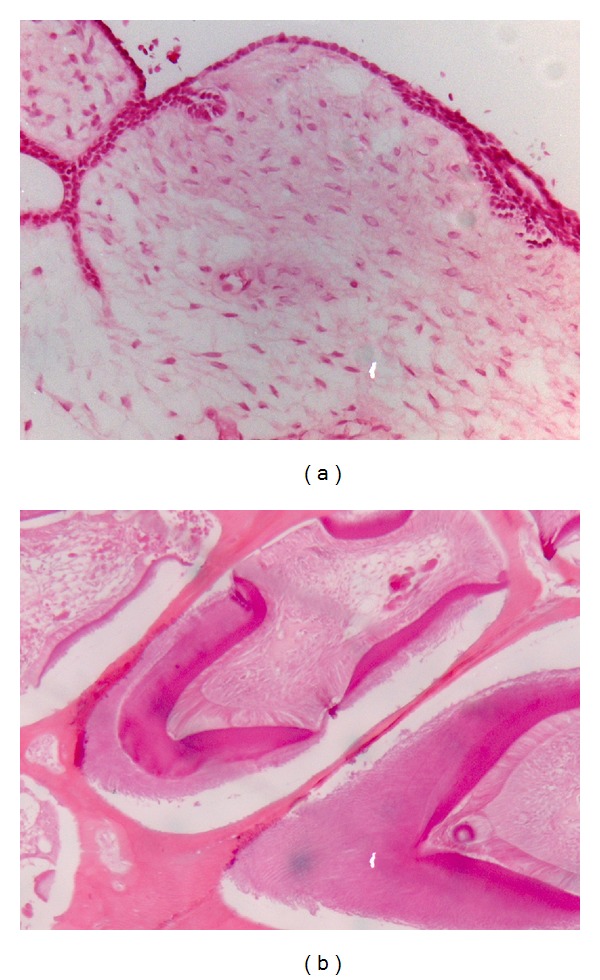
Low-magnification photomicrograph demonstrates that the lesion was composed of two patterns. (a) With areas of cell-rich mesenchymal stroma, cords, and follicles of odontogenic epithelium, consistent with ameloblastic fibroma. (b) A conglomerate of enamel, dentine, rudimentary small teeth, and cementum, consistent with complex odontoma.

Under general anesthesia, the second mandibular primary molar was extracted and the lesion attached to the permanent molar was enucleated with curettage of the bony walls. The bone was smoothed and the excisional site was sutured. The lesion, including the permanent molar and the primary molar, was submitted for pathologic examination. Microscopically, the lesion was composed of two patterns. One with areas of cell-rich mesenchymal stroma, cords, and follicles of odontogenic epithelium, consistent with ameloblastic fibroma. The second pattern was a conglomerate of enamel, dentine, rudimentary small teeth, and cementum, consistent with complex odontoma (Figure 3). The cumulative findings were compatible with characteristic AFO.

Following enucleation, a fresh sample of the lesion was also examined by classic cytogenetic analysis, as previously described [4–7]. The tissue was minced and cultured in RPMI-1640 medium, supplemented with antibiotics, glutamine, and 10% FCS and incubated at 37°C in 5% CO_2_ humidity. Cells were fixed after 3 and 8 days of culture and analyzed following standard procedures. More than 25 metaphases were analyzed on G-banded slides, and the karyotype was described according to the ISCN guidelines [8]. The cytogenetic workup revealed normal karyotype.

**Figure 4:**
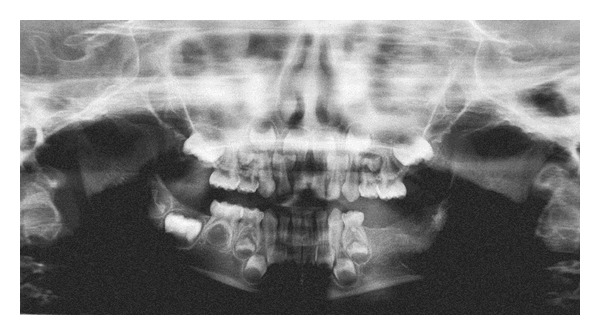
Postoperative panoramic radiograph, demonstrating that the radiodensity of the lesion area is similar to normal bone, indicating complete bone regeneration.

The postoperative course was uneventful. The last followup visit was approximately 1 year postoperative. Postoperative panoramic radiograph demonstrated that the radiodensity of the lesion area is similar to normal bone, indicating complete bone regeneration (Figure 4). The parents were informed of the low lesional recurrence rate.

## 3. Discussion

AFO is a mixed OT most commonly found in the posterior mandible and maxilla. It is part of a group of lesions defined as mixed odontogenic tumors, which include ameloblastic fibroma (AF), AFO, odontoma (ODO), and odontoameloblastoma [9]. These lesions are defined by the WHO as tumors composed of proliferating odontogenic epithelium embedded in cellular ectomesenchymal tissue that resembles dental papilla, with varying degrees of inductive changes and dental hard tissue formation. There is a debate among authors whether AFO is in fact a neoplasm or just a stage in the development of odontoma. Some authors [10, 11] have suggested that ameloblastic fibroma and AFO represent various stages of the same lesion, and that it will mature over time resulting ultimately in the formation of an ODO. On the other hand, the WHO classification considers that the ameloblastic fibroma, AFO, and ODO are different entities. Several residual or recurrent cases of AF have shown no evidence of further maturation into a more differentiated odontogenic lesion, such as AFO or ODO. Also, AFO affects patients with lower mean age than the AF, and the ultrastructural and immunohistochemical findings allow distinction between AF and AFO [1].

AFO is most common in children and young adults in the first and second decades, often found in the molar area of the mandible and maxilla, with no gender predilection. The most common clinical manifestations are local jaw expansion and failure of tooth eruption [12].

The differential diagnosis of AFO should include jaw lesions with mixed radiographic patterns, such as ODO, calcifying epithelial odontogenic tumor, calcifying odontogenic cyst, adenomatoid odontogenic tumor, and ameloblastic fibrosarcoma or ameloblastic fibroodontosarcoma.

Histologically, the specimen in this case was characteristic of an AFO, which has the same features as an AF with the addition of odontogenic hard tissues [13].

Cytogenetic analysis appeared to be normal in the present case. This is in contrast to chromosomal aberration such as loss of heterozygosity at chromosome region 9p22-p13 that was found in one sample out of three, paraffin-embedded tissue samples of AFO [14]. The reason for the normal karyotype in our patient versus the reported loss of heterozygosity in one other case, is unclear. It might be due to a difference in tumor grade at the time of the in vitro cell line analysis or due to some site-specific etiology. Further cytogenetic studies may shed more light on this issue in the future.

The management of an AFO involves enucleation and curettage, as most lesions easily separate from the surrounding bone [15]. Since most lesions are coronal to an unerupted or displaced tooth, the deciduous tooth is usually extracted along with the tumor. When associated with a permanent tooth, if possible, the tumor is enucleated allowing the involved permanent tooth to erupt. If the tumor is encasing the permanent tooth, as in the present case, both the tumor and the tooth should be removed to minimize recurrences.

AFO recurrence is usually associated with inadequate surgical removal or if tumor remnants persist in the enucleation margins. These tumor remnants can become either a recurrence or a malignant transformation.

Transformation of AFO to an ameloblastic fibrosarcoma or ameloblastic fibro-odontosarcoma is rare but has been reported [16–18]. The transformation can occur despite the normal karyotype. Similar observation was also reported in another type of OT, cystic jaw lesions, that have normal karyotype and and can undergo transformation to squamous cell carcinoma [19, 20]. 

AFO is a childhood tumor affecting the mandible and maxilla. It frequently causes expansion of the jaw with an effect on adjacent dentition. Conservative surgery is the treatment of choice. As malignant transformation is possible, long-term followup is recommended.
